# Patient factors that influence clinicians’ decision making in self-management support: A clinical vignette study

**DOI:** 10.1371/journal.pone.0171251

**Published:** 2017-02-06

**Authors:** Irene D. Bos-Touwen, Jaap C. A. Trappenburg, Ineke van der Wulp, Marieke J. Schuurmans, Niek J. de Wit

**Affiliations:** 1 University Medical Center Utrecht, Julius Center for Health Sciences and Primary Care, Utrecht, The Netherlands; 2 VUMC, EMGO Research Institute, Amsterdam, The Netherlands; University of the West Indies Faculty of Medical Sciences Mona, JAMAICA

## Abstract

**Background and aim:**

Self-management support is an integral part of current chronic care guidelines. The success of self-management interventions varies between individual patients, suggesting a need for tailored self-management support. Understanding the role of patient factors in the current decision making of health professionals can support future tailoring of self-management interventions. The aim of this study is to identify the relative importance of patient factors in health professionals’ decision making regarding self-management support.

**Method:**

A factorial survey was presented to primary care physicians and nurses. The survey consisted of clinical vignettes (case descriptions), in which 11 patient factors were systematically varied. Each care provider received a set of 12 vignettes. For each vignette, they decided whether they would give this patient self-management support and whether they expected this support to be successful. The associations between respondent decisions and patient factors were explored using ordered logit regression.

**Results:**

The survey was completed by 60 general practitioners and 80 nurses. Self-management support was unlikely to be provided in a third of the vignettes. The most important patient factor in the decision to provide self-management support as well as in the expectation that self-management support would be successful was motivation, followed by patient-provider relationship and illness perception. Other factors, such as depression or anxiety, education level, self-efficacy and social support, had a small impact on decisions. Disease, disease severity, knowledge of disease, and age were relatively unimportant factors.

**Conclusion:**

This is the first study to explore the relative importance of patient factors in decision making and the expectations regarding the provision of self-management support to chronic disease patients. By far, the most important factor considered was patient’s motivation; unmotivated patients were less likely to receive self-management support. Future tailored interventions should incorporate strategies to enhance motivation in unmotivated patients. Furthermore, care providers should be better equipped to promote motivational change in their patients.

## Background

In the Netherlands, a third of the older population has one or more chronic diseases [1]. Adequate self-management behaviour may be advantageous for individuals living with chronic disease [2]. In the guidelines for many chronic diseases, self-management support is included to help the patient “manage the symptoms, treatment, physical and psychosocial consequences and lifestyle changes inherent to living with a chronic condition” [3]. Chronic care is increasingly embedded in primary care. In addition, in the past decade, care provision has shifted from the general practitioner (GP) to the practice nurse (PN), especially for type 2 diabetes, chronic obstructive pulmonary disease (COPD) and cardiovascular disease [4].

Although self-management support is now an integral part of guidelines and promising results of self-management interventions have been observed, self-management interventions are not effective in all patients [5–7]. This implies that one-size-fits-all interventions are only successful in subgroups of patients. Tailoring self-management to the specific needs of a patient is expected to improve their efficacy for self-management [8] and therefore improve the effect size of interventions.

To move towards more tailored solutions knowledge is required on what patient factors are currently taken into account by care providers in providing self-management support. Little is known regarding how GPs and nurses make implicit decisions, such as whether to provide self-management support. In a previous survey study, we investigated which patient factors were considered important by care providers for the success of self-management; we found that motivation, knowledge of disease, educational level, self-efficacy and patient-provider relationship were the factors most frequently considered important [9]. In previous qualitative studies, nurses have explicitly stated that patient factors, such as motivation and capacity, affect their decision to provide self-management support [10, 11]. However, the relative importance of these factors in the actual decision-making process remains unknown. As mentioned by Ludwick et al., clinician insight into their decision-making processes is often limited [12]. Simulating clinical practice can be an effective way of determining how decisions are made in clinical practice.

The aim of this clinical vignette study is to investigate the relative importance of patient factors in the decision to provide self-management support as well as in the expectation that self-management support will be successful in a given patient. As PNs are becoming responsible for an important part of chronic care, differences in decision making between GPs and PNs are also explored.

## Methods

### Design

This is a cross-sectional study with a factorial survey design in which respondents are asked to evaluate clinical vignettes [13]. A vignette is a brief, written case history of a fictitious patient that is based on a realistic clinical situation [14]. Vignettes have been found to be a valid tool for simulating clinical practice and for measuring the quality of care in clinical practice [15]. In our vignettes the factors of interest (i.e., the patient factors) were varied in a systematic way. This was carried out by making random combinations between categories of factors within each vignette, therefore, vignettes with different case scenarios were created to explore the decision making and importance of each factor. Subsequently, GPs and PNs were presented with a sample of vignettes and answered questions that explored what they would decide to do when presented with the actual patient (S1 Questionnaire).

### Participants

The study population consisted of general practitioners (GPs), last-year GP trainees and practice nurses (PNs) working in general practice in the Netherlands. Last-year GP trainees were included because we hypothesised that younger, recently trained clinicians would consider different patient factors to influence their decision-making. Potential participants were approached through regional primary health care organisations, the General Practice Specialty Training institutes in the Netherlands, and by mail from a national PN network.

### Data collection

Data were collected through an online questionnaire. Participants were recruited from March – June 2014 for a prior survey, in which participants were asked whether they could be approached for a follow-up study; after providing consent, they were asked for their email addresses. From July –October 2014, the clinical vignette survey was completed. Three reminders were sent in the event of non-response.

The survey consisted of 12 clinical vignettes in which eleven patient factors were systematically incorporated. Since definitions and perceptions of what self-management support entails can vary substantially between professionals, we defined self-management support as follows: *Self-management support consists of a transfer of information and a minimum of two of the following components*: *active stimulation of symptom monitoring*, *medication management*, *education in problem-solving skills (*i.e., *self-treatment such as managing acute exacerbations*, *utilising resources and managing stress/symptoms) and enhancement of medication adherence*, *physical activity*, *dietary intake or smoking cessation* [16].

GPs and PNs were instructed to read each vignette as if it were an actual patient consultation, and they were asked to rate the likelihood that they would provide self-management support to the patient in question on a 5-point Likert scale (very unlikely—very likely)[17, 18]. Subsequently, they were asked how successful they thought that self-management support would be in this patient. The response options were categorised on a 5-point Likert scale, from not at all successful to very successful.

### Selection of patient factors

The literature was comprehensively searched for potential patient factors that could influence patients’ self-management capacity, and these factors were discussed within the research team, which consisted of experts in nursing, general practice, and self-management. A selection of 15 factors that the research team thought were clinically relevant and of potential influence on the decision to provide self-management support were presented in a survey to GPs and PNs. The participants were asked which patient factors they considered important for the success of self-management and whether there were any missing factors in the list provided. This validation step confirmed the 15 factors identified from the literature. Illness perception and depression were not described and not validated in the survey; however, in our recent study [19], these factors were found to be associated with activation for self-management. Therefore, we decided to add illness perception and anxiety or depressive disorders to the list. Subsequently, we aimed to reduce our list of 17 patient factors for our final selection because readers cannot process that extent of information in a vignette and because vignettes should represent clinical practice in the number and complexity of factors [20]. Therefore, we weighted the factors based on their importance in the survey study and in the literature and discussed possible overlap of the factors in an expert meeting, resulting in a reduced list of 11 factors (Table 1). Level of autonomy was considered to be a prerequisite for self-management and therefore too obvious a factor. Socioeconomic status was considered to overlap with education level, and ethnicity too varied, as some practices worked with underrepresented populations. Co-morbidities and private problems were considered too imprecise and to partly coincide with depressive and anxiety disorders. Gender was rarely considered to be of influence on the success of self-management in the previous questionnaire, which is in line with two recent individual patient data meta-analyses [21, 22]. We chose the two most prevalent chronic conditions that are managed by both GPs and PNs in the Netherlands, namely COPD and diabetes mellitus (DM).

**Table 1:** Factors and categories included in the vignettes.

*Patient Factors*	*Categories (Levels)*	*Definition:*
Age	40 years old 60 years old 80 years old	N.A.
Education level	High education level Medium education level Low education level	College or university degree
		Secondary school or vocational training
		Primary school through vocational training
Disease	COPD DM II	Chronic obstructive pulmonary disease
		Diabetes Mellitus type 2
Knowledge of disease	Has sufficient knowledge of the disease Has insufficient knowledge of the disease	N.A.
Illness perception	Having a realistic illness perception Not having a realistic illness perception	The patient has/does not have a realistic perception about the disease, cause, timeframe, consequences or treatment of the disease
Disease burden	No symptoms	COPD: Virtually no dyspnoea, no exacerbations and no reduced exercise tolerance
		DM II: Stable and well controlled diabetes (no glucose fluctuations (hyper/hypo) or diabetic-related complications such as diabetic foot, peripheral neuropathy, ulcers, retinopathy)
	Mild symptoms	COPD: Dyspnoea only during strenuous exercise AND/OR incidental exacerbations AND/OR mildly reduced exercise tolerance
		DM II: Slightly unstable diabetes (rare to occasional glucose fluctuations (hyper/hypo) AND/OR early-stage/mild diabetic-related complications such as diabetic foot, peripheral neuropathy, ulcers, retinopathy)
	Severe symptoms	COPD: Dyspnoea during mild exercise AND/OR frequent exacerbations AND/OR strongly reduced exercise tolerance
		DM II: Unstable or poorly controlled diabetes (severe glucose fluctuations (hyper/hypo) AND/OR more advanced diabetic-related complications such as diabetic foot, peripheral neuropathy, ulcers, retinopathy)
Social support	Social support at home No social support at home	Having assistance or comfort from other people to cope with a variety of problems. By home, we mean people who come to a patient’s home (e.g., partner, family members, neighbours, friends)
Self-efficacy	Sufficient self-efficacy Low self-efficacy	Has a sufficient/low belief in one's own ability to complete tasks and reach goals
Motivation	Motivated for self-management Not motivated for self-management	Presence/absence of a state or condition of being motivated or having a strong reason to act or accomplish something
Anxiety or depressive disorder	Present Absent	N.A.
Patient-provider relationship	Good patient-provider relationship Poor patient-provider relationship	Having/not having a good, safe and adequate relationship between the carer and patient based on mutual trust and respect

We defined categories for each of the patient factors. As it was preferred to provide only a few categories to maintain a small total number of possible vignettes, we restricted each factor to 2 or 3 categories. Our final selection of factors and categories is shown in Table 1.

### Selection of vignettes

The full factorial design combined all the levels of one factor with all the levels of the other factors. With the selected factors and factor levels, 6912 vignettes (2^8^*3^3^) were created. As completing all of these vignettes would be too burdensome for the respondents, the vignettes had to be efficiently selected (i.e., a fractional factorial design). Prior to selecting the fractional factorial design, unrealistic vignettes were removed from the full factorial design. These included vignettes representing patients with insufficient knowledge of their illness but with a realistic illness perception and vignettes representing patients 40 years of age who suffered from COPD. In R Cran for Windows, using the AlgDesign package, 96 vignettes were selected by applying the Federov Algorithm [23, 24]. We decided to only account for main effects in the analyses, resulting in an explained variance between 70% and 90% [25]. The quality of the resulting design was evaluated by means of the G efficiency parameter, which ranges from 0 (inefficient design) to 1 (efficient design) [24]. The resulting design had a G efficiency of 0.96 and was therefore considered efficient. The selected vignettes were subsequently allocated into eight blocks, each comprising 12 vignettes. By dividing vignettes into blocks, we ensured that the main effects could be estimated within each block. The loss of efficiency due to blocking appeared to be limited, as the geometric mean of efficiency was 0.996.

The sample size was based on the assumption that 6 respondents per vignette would be required to reliably perform the analyses and retrieve valid results [25]. Since we aimed to perform the analyses separately for GPs and PNs and had 8 blocks of vignettes, we needed (8*6) 48 GPs and 48 PNs. Each care provider received a randomly allocated set of 12 vignettes. The randomisation procedure was stratified by type of primary care provider (GP, PN).

### Case description of clinical vignette

The vignettes were written by one of the research team members and subsequently discussed in the research team to ensure that the vignette was as realistic as possible. The vignettes were piloted in n = 6 clinicians (n = 3 GPs, n = 3 nurses) to evaluate whether the vignettes were clear and represented realistic patients. An example of a vignette is shown in Box 1.

**Box 1. Example of a clinical vignette used in the survey**

Patient X is **40 years old**, has a **medium education level** and comes to see you for a **diabetes checkup**. The patient currently experiences **no diabetes-related symptoms**. You do **not experience a good patient-provider relationship**. The patient has **sufficient knowledge of the disease** and has a **realistic illness perception**. The patient **has social support at home**. The patient is **not motivated for self-management** and has l**ow self-efficacy for self-management**. The patient does **not have an anxiety or depressive disorder**.

### Data analyses

Descriptive statistics were used to describe the respondent characteristics and were performed in SPSS version 22.0 for windows [26]. Subsequent analyses were conducted using R-cran for windows [23]. The associations between the respondent’s choices and patient factors were explored by means of mixed ordered logit regression. This model was chosen because our outcome variables were ordinal and each respondent evaluated 12 vignettes, and their answers were therefore clustered. The model was created by simultaneously entering all factors into the model. This approach was selected because the respondents were also presented with 11 factors at one time. The model accounted for the panel dimension of the data (respondents completed 12 vignettes each) by including respondent number as a random effect. The main model assumption, which was the assumption of proportional odds, was assessed graphically by constructing line graphs of all the variables and reviewing them for parallelism (S1 Fig). Only some small deviations were seen in the lower or upper lines which were caused by a very small number of participants representing that line. Therefore, our conclusion was that the proportional odds assumption was met (equal slopes, parallel lines). The model was subsequently used as the basis for determining the relative importance of the factors. Using partial log-likelihood analyses, the difference in log-likelihood of the main effects model was compared with those of models in which one factor was removed. Finally, 95% confidence intervals of the relative importance measures in GPs and PNs were calculated with bootstrapping (n = 1000).

## Results

Of the 230 health care providers approached, 161 agreed to participate (response rate of 70.0%). Of the 1932 vignettes that were presented, 1762 were completed (91.2%), and 21 (13%) of 161 respondents had incomplete data. Respondents with missing values and those without missing values did not differ in terms of profession, practice type, gender, age, work experience or in their perceptions (role, aim and importance) of self-management.

As respondents with missing values could not see the next vignette if they did not complete the previous one, the missing values were not linked to the factors. There was sufficient statistical power to perform the analyses with the complete cases; accordingly, we chose not to impute the data but to perform complete case analyses. We performed our analyses with 60 GPs and 80 PNs. Their baseline characteristics are shown in Table 2.

**Table 2:** Baseline characteristics of participants.

	*General practitioner (n = 60)*	*Practice nurse (n = 80)*
**Gender**		
Male	30 (50%)	2 (2.5%)
Female	30 (50%)	78 (97.5%)
**Age**	47.1 ± 11.7	45.8 ± 10.5
**Years in function**	15.6 ± 10.5	7.1 ± 4.4
**Practice type**		
Single	16 (26.7%)	21 (26.3%)
Dual	24 (40.0%)	27 (33.8%)
Group	20 (33.3%)	32 (40.0%)
**Practice location**		
Large city (>100,000 citizens)	18 (30.0%)	14 (17.5%)
City (30,000–100,000 citizens)	18 (30.0%)	30 (37.5%)
Small city (10,000–30,000 citizens)	16 (26.7%)	26 (32.5%)
Small town (<10,000 citizens)	8 (13.3%)	10 (12.5%)
**Received training in motivational interviewing**	14 (23.3%)	60 (75%)

### Relative importance of patient factors

**Fig 1:**
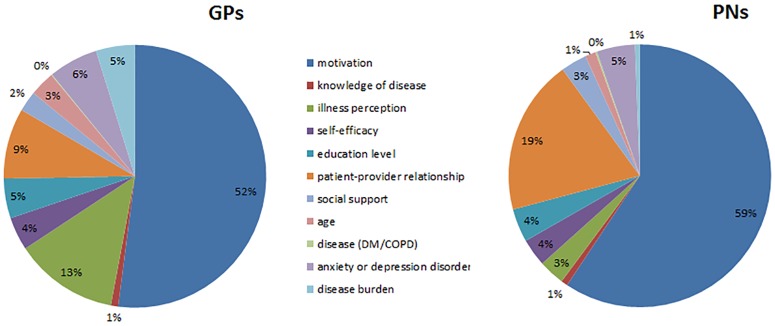
Relative importance of patient factors in providing self-management. DM = Diabetes Mellitus, COPD = Chronic Obstructive Pulmonary Disease.

**Fig 2:**
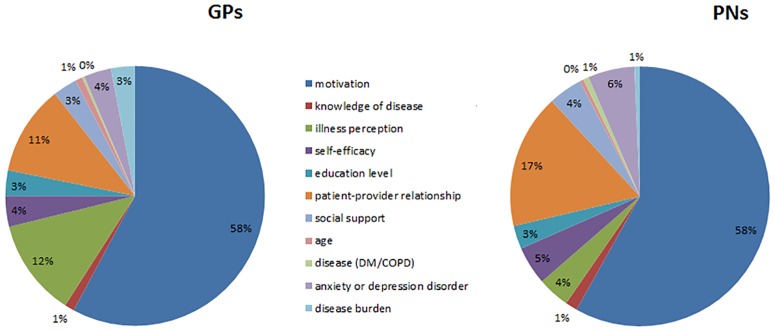
Relative importance of patient factors in judging whether a patient will be successful in self-management. DM = Diabetes Mellitus, COPD = Chronic Obstructive Pulmonary Disease.

Self-management support was considered unlikely to be provided in 29% of the vignettes, and in 46% of the vignettes, this support was expected to have a low chance of success. The relative importance of the factors is shown in Figs 1 and 2 (a table containing the confidence intervals is available in the S1 Table). The analysis of the relative importance of each attribute showed that motivation was by far the most important factor in the likelihood of providing self-management support (GPs 52%, PNs 59%) as well as in their judgement of how successful self-management support would be (GPs and PNs 58%). For GPs, illness perception was the next most important factor, with 13%, while for PNs, the patient-provider relationship was the next most important factor, with 19%. Type of disease, disease burden, knowledge of disease, and age were considered relatively unimportant factors in both the decision to provide self-management and in the estimation of the likelihood of success.

### Importance of the categories

The odds ratios (ORs) of the factor categories for the decision to provide self-management support are presented in Table 3. The OR represents the average odds that self-management would likely be provided given the factor category compared to the average odds that self-management would be provided in the absence of that category. Thus, the OR of a GP providing self-management support when a patient was motivated was, on average, 8.34 times higher compared to the odds for when a patient was not motivated. Except for the factor ‘type of disease’, all factors showed a positive OR and were likely to be associated with a higher OR of receiving self-management support compared with the OR in the absence of the factor. Overall, there were no significant differences between GPs and PNs in the contribution of factors; motivation showed the highest OR for both groups. GPs had a higher OR for illness perception, though this difference was not statistically significant. Furthermore, PNs more often had an OR of 1 in their confidence interval.

**Table 3:** Odds ratios of the categories for providing self-management support to a patient—based on examination of 64 vignettes by 60 general practitioners and 80 practice nurses.

	*General practitioner Odds ratio (CI)*	*Practice nurse Odds ratio (CI)*	*Total Odds ratio (CI)*
Age			
40 years vs. 80 years	1.94 (1.28–2.93)	1.54 (1.08–2.20)	1.68 (1.28–2.19)
60 years vs. 80 years	1.42 (1.02–1.99)	1.17 (0.88–1.56)	1.25 (1.01–1.56)
Education level			
Medium vs. low	1.61 (1.13–2.30)	1.24 (0.92–1.68)	1.37 (1.09–1.73)
High vs. low	2.12 (1.47–3.04)	1.89 (1.39–2.57)	1.95 (1.55–2.46)
Disease DM-II vs. COPD	1.08 (0.77–1.50)	1.15 (0.87–1.54)	1.12 (0.90–1.39)
Sufficient knowledge of disease vs. insufficient knowledge of disease	1.38 (0.97–1.97)	1.34 (0.99–1.81)	1.34 (1.07–1.69)
Realistic illness perception vs. non-realistic illness perception	3.41 (2.36–4.92)	1.77 (1.30–2.39)	2.30 (1.83–2.90)
Disease burden			
Mild symptoms vs. no symptoms	1.86 (1.30–2.67)	1.05 (0.78–1.43)	1.32 (1.05–1.67)
Severe symptoms vs. no symptoms	1.97 (1.37–2.82)	1.27 (0.93–1.72)	1.51 (1.20–1.90)
Social support vs. lack of social support	1.55 (1.16–2.08)	1.60 (1.25–2.05)	1.58 (1.31–1.91)
Sufficient self-efficacy vs. low self-efficacy	1.76 (1.31–2.37)	1.63 (1.27–2.10)	1.66 (1.37–2.00)
Motivated for self-management vs. not motivated for self-management	8.34 (5.99–11.6)	8.54 (6.44–11.33)	8.33 (6.73–10.31)
No depression or anxiety disorder vs. depression or anxiety disorder	1.99 (1.48–2.67)	1.76 (1.37–2.26)	1.83 (1.51–2.21)
Good patient-provider relationship vs. poor patient-provider relationship	2.28 (1.69–3.08)	3.21 (2.48–4.16)	2.75 (2.27–3.35)

CI = confidence interval, DM-II = Diabetes mellitus type II, COPD = Chronic obstructive pulmonary disease

Table 4 shows the ORs for when self-management was considered to be successful in a patient based on the presence of a certain factor. Motivation for self-management vs non-motivation showed the highest ORs (GPs OR 16.1, PNs OR 11.0). A statistically significant difference between GPs and PNs was found for illness perception (GPs OR 4.1 (CI 2.8–6.0), PNs OR 2.0 (CI 1.5–2.8)). Overall, the odds ratios were higher when considering whether self-management would be successful than when the GPs reported the likelihood of providing self-management. The exceptions to this trend included education level, in which the OR remained more or less the same, and age and disease severity, in which the ORs decreased and the association was therefore less strong.

**Table 4:** Odds ratios of the categories for expecting self-management support to be successful in a patient—based on examination of 64 vignettes by 60 general practitioners and 80 practice nurses.

	*General practitioner Odds ratio (CI)*	*Practice nurse Odds ratio (CI)*	*Total Odds ratio (CI)*
Age			
40 years vs. 80 years	1.54 (1.01–2.33)	1.29 (0.91–1.85)	1.37 (1.05–1.80)
60 years vs. 80 years	1.13 (0.80–1.60)	1.21 (0.91–1.62)	1.17 (0.94–1.47)
Education			
Medium vs. low	1.55 (1.07–2.23)	1.46 (1.07–1.99)	1.49 (1.18–1.88)
High vs. low	2.06 (1.42–2.97)	1.83 (1.34–2.50)	1.91 (1.51–2.41)
Disease DM-II vs. COPD	1.25 (0.88–1.76)	1.32 (0.98–1.77)	1.30 (1.04–1.62)
Sufficient knowledge of disease vs. insufficient knowledge of disease	1.57 (1.09–2.27)	1.54 (1.13–2.11)	1.56 (1.23–1.97)
Realistic illness perception vs. non-realistic illness perception	4.13 (2.83–6.01)	2.04 (1.50–2.78)	2.73 (2.15–3.46)
Disease burden			
Mild symptoms vs. no symptoms	1.59 (1.11–2.30)	1.28 (0.94–1.75)	1.40 (1.10–1.77)
Severe symptoms vs. no symptoms	2.02 (1.40–2.92)	1.33 (0.97–1.81)	1.57 (1.24–1.99)
Social support vs. lack of social support	1.79 (1.32–2.41)	1.82 (1.41–2.35)	1.80 (1.48–2.19)
Sufficient self-efficacy vs. low self-efficacy	1.91 (1.41–2.59)	1.91 (1.48–2.27)	1.91 (1.57–2.32)
Motivated for self-management vs. not motivated for self-management	16.07 (11.10–23.29)	11.02 (8.20–14.81)	12.84 (10.19–16.16)
No depression or anxiety disorder vs. depression or anxiety disorder	1.85 (1.37–2.50)	2.04 (1.58–2.64)	1.95 (1.60–2.36)
Good patient-provider relationship vs. poor patient-provider relationship	3.10 (2.27–4.22)	3.42 (2.63–4.46)	3.22 (2.63–3.93)

CI = confidence interval, DM-II = Diabetes mellitus type II, COPD = Chronic obstructive pulmonary disease

## Discussion

This study found that in both types of care providers, motivation was the most important factor in deciding to provide self-management support and in expecting self-management support to be successful. For GPs, illness perception was the next most important factor, and for PNs, the patient-provider relationship was the next most important. All other factors played either a minor or no role in providers’ decision to provide self-management support. Furthermore, this study showed that it was unlikely that self-management support would be provided in every patient, meaning that not every patient was considered suitable for self-management and that certain patients would not receive self-management support.

This final conclusion supports the importance of understanding which factors play a role in providers’ decision making regarding self-management support. From previous research, we did expect that motivation would play an important role in self-management support. In a qualitative study, nurses mentioned that motivation and capability were the most important factors in self-management support [11], and in the survey preceding this study, motivation was often mentioned as a factor [9]. We now understand the importance of motivation and that when motivation is not present, it is unlikely that self-management support will be provided.

This finding raises the question of whether it is justifiable to not provide self-management support to patients who are unmotivated or whether attempts to provide this support should at least be made. According to the transtheoretical model of behaviour change, people need to be guided through several stages of change to increase their motivation before they are ready for action [27]. However, as motivation is such a huge barrier to self-management, perhaps too much importance has been placed on motivation, and health care providers thus find it difficult to identify what causes a patient to be unmotivated. According to the Health Belief Model, perceived susceptibility to and severity of illness, perceived costs and benefits, and self-efficacy can motivate a person to engage or not engage in health-related behaviour [28]. In The Behaviour Change Wheel, behaviour is construed as a product of capability, opportunity and motivation [29]. These constructs interact within what can be conceptualized as a system. Assuming that we have the opportunity to perform a variety of behaviours and the capability, motivation is the part of the system that determines what we actually do and how we do it. Many more motivational and behavioural change theories exist; however, the key is that motivation does not function autonomously, and as Rollnick and colleagues state that “no person is completely unmotivated” [30], ambivalence towards behaviour should be explored. Motivational interviewing aims to explore this ambivalence and is defined as “a directive, client-centred counselling style for eliciting behaviour change by helping clients to explore and resolve ambivalence”[30]. Many care providers in the Netherlands have taken courses in motivational interviewing (MI); however, studies show that using MI during routine consultations in primary care is not standard practice (yet), despite having followed MI courses [31, 32]. MI is not easily learned or mastered. It requires a substantial amount of skill and involves the conscious and disciplined use of specific communication principles and strategies to evoke the person’s own motivations for change [33]. This approach is in line with our results, since motivation was the most important factor determining the likelihood of providing self-management, and when there is a lack of motivation, it is unlikely that self-management support will be provided. It was evident that care providers do not know how to cope with unmotivated patients. This would require more intense training of GPs and PNs in motivational interviewing.

GPs also considered illness perception an important factor. Illness perceptions (ideas/beliefs that patients have about their illness and symptoms) can vary substantially between patients and are important for establishing coping behaviours and illness-specific behaviours such as adherence to treatment [34, 35]. The literature suggests that illness perception is important; however, PNs did not seem to consider it a very important factor. On the other hand, PNs considered the patient-provider relationship to be quite an important factor. The most mentioned factors that influence this relationship were mutual trust, confidence in each other and good communication. In a previous study, nurses also mentioned that building a strong relationship and mutual trust helped patients feel more comfortable sharing personal information, and nurses indicated that patients with involved and compassionate nurses would more readily improve their self-management behaviour [36]. In motivational interviewing, communication and listening with empathy are prerequisites for behaviour change [30]. Nurses in our study were predominantly female, but also have completed more motivational interview courses than GPs, which might have led to increased awareness of the importance of the patient-provider relationship, although the difference in relative importance is not significant between GPs and nurses. Nurses may also be more aware of the patient-provider relationship because patients choose their GP but not their nurse. We also hypothesised that care providers may find it easier to promote responsibility in a patient with whom they have a good relationship than in patients with whom they do not have a good relationship. GPs considered illness perception of more importance than nurses. One explanation of this finding might be the difference in practice training. In GP training, much attention is paid to communication (often provided by psychologists), and this emphasis could lead to more awareness of the importance of illness perception. Although we cannot draw conclusions regarding why GPs considered illness perception more important while PNs emphasised the patient-provider relationship, both factors can be influenced by exploring illness perceptions and by maintaining open attitudes towards patients and good communication skills.

A remarkable finding was that knowledge of disease was not a decisive factor at all. In the previous survey study, professionals ranked knowledge of disease as the second most influential factor for successful self-management; however, this characteristic was not found to have an influence in the vignettes. Furthermore, self-efficacy was also a minor factor in this study, while in the previous survey, it was ranked the fourth most important factor. The well-known self-management programme Chronic Disease Self-Management Program (CDSMP) incorporates strategies suggested by Bandura to enhance self-efficacy to help patients become more confident and knowledgeable [37]. In addition, many other self-management programmes aim to increase knowledge of disease and treatment, as well as self-efficacy. In this study, care providers did not consider knowledge of disease and self-efficacy to be important factors. A possible explanation might be that in contrast to the other factors in the vignettes, both knowledge and self-efficacy can be influenced and to a lesser degree are seen as barriers towards success of self-management. Based on commonly used theoretical frameworks such as the social cognitive theory [38], both knowledge acquisition and improvement of self-efficacy are key elements in many self-management interventions [39–41]. Particularly self-efficacy has shown to be an important mediator in the causal mechanism towards success of self-management.

### Strengths and limitations

To our knowledge, this is the first study to explore the relative importance of certain patient factors in decision making concerning self-management support. Our sample size was sufficient to answer the research questions. We used an efficient design to explore patient factors in decision making. In our clinical vignettes, the factors were rather explicit, whereas more nuances can be expected in clinical practice. Although this is a limitation of vignettes, this approach more clearly shows the relative importance of factors. We can only draw conclusions regarding the relative importance of the factors, and we do not know why care providers selected them; however, the findings do provide insight into decision making. We could not account for all possible patient factors because that would have made the vignettes unreadable and because readers cannot simultaneously consider that many factors. The findings are therefore based on our current selection of factors; however, there could be other important factors in decision making concerning self-management support that we did not include in this study. For example: apart from assessing the relative importance of emotional disorders (depression and anxiety), the vignettes largely obscured the influence of multi-morbidity. Future research is needed to evaluate the relative importance of variations in both the amount and specific patterns in comorbidities.

## Conclusions

This study shows that patient factors play a role in the decision to provide self-management support, as in 30% of the presented cases, self-management support was unlikely to be provided. By far, the most important factor was motivation in both the likelihood of providing self-management as well as in expecting that self-management support would be successful for a patient. Unmotivated patients were less likely to receive self-management support. Future tailored interventions should incorporate strategies that enhance motivation in unmotivated patients. Furthermore, care providers need more insight into what causes a patient to be unmotivated, what other factors play a role and how to increase motivation. Increasing providers’ motivational interviewing skills could be a strategy to improve their ability to stimulate motivational change. Few differences between GPs and PNs were observed, although GPs placed more importance on illness perception and PNs on the patient-provider relationship. Scientific efforts are needed to evaluate whether this is caused by differences in roles or (vocational) training. All other factors played a minor or even no role in self-management support. The three patient factors that were the most important are all factors that can be influenced by care providers by increasing their awareness, communication skills and probably consultation time.

In conclusion, in current primary care practice, motivation is perceived to be the most important factor in deciding to provide self-management support and in expecting that self-management support will be successful.

## Supporting information

S1 Questionnaire An example of a questionnaire in English and in Dutch. (DOCX)

S1 Fig Graphical test for proportional odds assumption. The lines in the graph represent the different dichotomized models (solid line represents the model unlikely to very likely to provide SMS vs. very unlikely to provide SMS, and the dashed line represents the model somewhat likely to very likely to provide SMS vs unlikely and very unlikely to provide SMS, etc., for the dotted and the dashed line).
(TIF)

S1 Table Relative importance of factors. SM = Self-management, CI = confidence interval, GP = general practitioner, PN = practice nurse, DM-II = Diabetes mellitus type II, COPD = Chronic obstructive pulmonary disease.
(DOCX)
