# Supplementary material for: Patient factors that influence clinicians’ decision making in self-management support: A clinical vignette study
Source: PLoS One. 2017 Feb 6;12(2):e0171251. doi: 10.1371/journal.pone.0171251 (PMC5293247; doi:10.1371/journal.pone.0171251)
Supplement: S1 Questionnaire — (DOCX) [file pone.0171251.s001.docx]

**Questionnaire**

In this questionnaire we present you with case descriptions. It is not about right or wrong. We want to ask you to form an image of the patient presented in the case description and to imagine that this patient is sitting in front of you in your consultation room and to subsequently answer the questions below the case description.

To be sure that everyone filling out this questionnaire has the same idea about self-management support we give the following definition of self-management support and we want to ask you to keep this definition in mind on answering the questions.

*Self-management support consists of a transfer of information and a minimum of two of the following components: active stimulation of symptom monitoring, medication management, education in problem-solving skills (*i.e*., self-treatment such as managing acute exacerbations, utilising resources and managing stress/symptoms) and enhancement of medication adherence, physical activity, dietary intake or smoking cessation.*

The answers provided by you will be used for scientific research. By filling out this questionnaire we presume your permission for using your answers for this purpose and we will anonymously use and analyse them.

Case description 1

Patient X is **80-years old**, has a **high education level** and comes to see you for a **COPD checkup**. The patient currently experiences **severe COPD related symptoms**. You **experience a good patient-provider relationship**. The patient has **sufficient knowledge of the disease** and has a **realistic illness perception**. The patient **has no social support at home**. The patient is **motivated for self-management** and has **sufficient self-efficacy for self-management**. The patient **has an anxiety or depressive disorder.**

1. How successful do you think self-management support will be in this patient? (This question is asked after every case description)

1 2 3 4 5

Not at all successful not successful somewhat successful successful very successful

1. How likely is it that you will support this patient in self-management? (This question is asked after every case description)

1 2 3 4 5

Very unlikely unlikely somewhat likely likely very likely

1. I am confident about my answers: (This question is asked after every case description)

1 2 3 4 5

Strongly disagree disagree neither agree nor disagree agree strongly agree

Case description 2

Patient X is **40-years old**, has a **medium education level** and comes to see you for a **diabetes checkup**. The patient currently experiences **no diabetes related symptoms**. You **experience a poor patient-provider relationship**. The patient has **sufficient knowledge of the disease** and has a **realistic illness perception**. The patient **has social support at home**. The patient is **not motivated for self-management** and has l**ow self-efficacy for self-management**. The patient does **not have an anxiety or depressive disorder.**

Case description 3

Patient X is **40-years old**, has a **high education level** and comes to see you for a **diabetes checkup**. The patient currently experiences **mild diabetes related symptoms**. You do **experience a good patient-provider relationship**. The patient has **sufficient knowledge of the disease** and has a **realistic illness perception**. The patient **has social support at home**. The patient is **motivated for self-management** and has **sufficient self-efficacy for self-management**. The patient does **not have an anxiety or depressive disorder.**

Case description 4

Patient X is **80-years old**, has a **low education level** and comes to see you for a **COPD checkup**. The patient currently experiences **mild COPD related symptoms**. You do **experience a good patient-provider relationship**. The patient has **sufficient knowledge of the disease** and has a **realistic illness perception**. The patient **has no social support at home**. The patient is **motivated for self-management** and has **low self-efficacy for self-management**. The patient does **not have an anxiety or depressive disorder.**

Case description 5

Patient X is **80-years old**, has a **low education level** and comes to see you for a **COPD checkup**. The patient currently experiences **no COPD related symptoms**. You **experience a poor patient-provider relationship**. The patient has **insufficient knowledge of the disease** and **does not have a** **realistic illness perception**. The patient **has social support at home**. The patient is **not motivated for self-management** and has **sufficient self-efficacy for self-management**. The patient does **have an anxiety or depressive disorder.**

Case description 6

Patient X is **60-years old**, has a **medium education level** and comes to see you for a **diabetes checkup**. The patient currently experiences **severe diabetes related symptoms**. You **experience a good patient-provider relationship**. The patient has **sufficient knowledge of the disease** and **has a** **realistic illness perception**. The patient **has no social support at home**. The patient is **motivated for self-management** and has **low self-efficacy for self-management**. The patient does **not have an anxiety or depressive disorder.**

Case description 7

Patient X is **60-years old**, has a **low education level** and comes to see you for a **COPD checkup**. The patient currently experiences **mild COPD related symptoms**. You **experience a good patient-provider relationship**. The patient has **insufficient knowledge of the disease** and **does not have a** **realistic illness perception**. The patient **has social support at home**. The patient is **not motivated for self-management** and has **sufficient self-efficacy for self-management**. The patient does **not have an anxiety or depressive disorder.**

Case description 8

Patient X is **80-years old**, has a **low education level** and comes to see you for a **diabetes checkup**. The patient currently experiences **mild diabetes related symptoms**. You **experience a good patient-provider relationship**. The patient has **insufficient knowledge of the disease** and **does not have a** **realistic illness perception**. The patient **has no social support at home**. The patient is **not motivated for self-management** and has **low self-efficacy for self-management**. The patient does **not have an anxiety or depressive disorder.**

Case description 9

Patient X is **60-years old**, has a **high education level** and comes to see you for a **diabetes checkup**. The patient currently experiences **severe diabetes related symptoms**. You **experience a poor patient-provider relationship**. The patient has **sufficient knowledge of the disease** and **has a** **realistic illness perception**. The patient **has social support at home**. The patient is **motivated for self-management** and has **low self-efficacy for self-management**. The patient **has an anxiety or depressive disorder.**

Case description 10

Patient X is **60-years old**, has a **medium education level** and comes to see you for a **COPD checkup**. The patient currently experiences **severe COPD related symptoms**. You **experience a good patient-provider relationship**. The patient has **sufficient knowledge of the disease** and **has a** **realistic illness perception**. The patient **has social support at home**. The patient is **not motivated for self-management** and has **low self-efficacy for self-management**. The patient **has an anxiety or depressive disorder.**

Case description 11

Patient X is **80-years old**, has a **low education level** and comes to see you for a **diabetes checkup**. The patient currently experiences **no diabetes related symptoms**. You **experience a good patient-provider relationship**. The patient has **insufficient knowledge of the disease** and **does not have a** **realistic illness perception**. The patient **has no social support at home**. The patient is **not motivated for self-management** and has **sufficient self-efficacy for self-management**. The patient does **have an anxiety or depressive disorder.**

Case description 12

Patient X is **60-years old**, has a **high education level** and comes to see you for a **COPD checkup**. The patient currently experiences **no COPD related symptoms**. You **experience a poor patient-provider relationship**. The patient has **insufficient knowledge of the disease** and **does not have a** **realistic illness perception**. The patient **has no social support at home**. The patient is **motivated for self-management** and has **sufficient self-efficacy for self-management**. The patient does **not have an anxiety or depressive disorder.**

**Voorbeeld vragenlijst**

In deze vragenlijst leggen wij u casuïstiek voor. Het gaat niet om goed en fout. Het is de bedoeling dat u zich een beeld vormt van de patiënt in de casus, dat u bedenkt dat die patiënt tegenover u zit in de spreekkamer en dan de 2 onderstaande vragen beantwoord.

Om zeker te weten dat iedereen hetzelfde idee van zelfmanagement ondersteuning heeft bij het beoordelen van de casuïstiek volgt hieronder een definitie van zelfmanagement ondersteuning. Houdt deze definitie in uw achterhoofd bij het invullen van de vragenlijst

*Zelfmanagement ondersteuning bestaat ten minste uit kennisoverdracht en een combinatie van minimaal twee van de volgende componenten: actief stimuleren van symptoommonitoring, medicatiemanagement, het maken van keuzes ten aanzien van zelfbehandeling of extra contact met zorgverleners, veranderen van leefstijl op het gebied van fysieke activiteit, voedingsgewoonten of rookgedrag.*

Onderstaande antwoorden zullen gebruikt worden voor wetenschappelijk onderzoek. Met het invullen van de vragenlijst geeft u toestemming voor deelname aan dit onderzoek en dat uw antwoorden anoniem gebruikt mogen worden voor wetenschappelijk onderzoek.

Casus 1

Patiënt X van **80 jaar, hoog opgeleid,** komt bij u op het spreekuur voor een **COPD** controle. De patiënt **ervaart hier ernstige symptomen van**. U heeft **een goede behandelrelatie met de patiënt**. De patiënt **heeft voldoende kennis van de ziekte** en heeft een **realistische ziekte-perceptie**. Er is **geen sociale steun thuis**. De patiënt **is gemotiveerd voor zelfmanagement**. De patiënt heeft **vertrouwen in eigen kunnen**. De patiënt heeft **een angst of stemmingsstoornis**.

Hoe succesvol denkt u dat bij deze patient zelfmanagement ondersteuning zal zijn

1 2 3 4 5

Helemaal niet succesvol niet succevol tamelijk succesvol succesvol zeer succesvol

Hoe waarschijnlijk denkt u dat het is dat u deze patiënt zelfmanagement ondersteuning aanbiedt?

1 2 3 4 5

Helemaal niet waarschijnlijk niet waarschijnlijk enigszins waarschijnlijk waarschijnlijk erg waarschijnlijk

Ik ben zeker van mijn antwoorden:

1 2 3 4 5

Volledig mee oneens mee oneens mee eens, noch mee oneens mee eens volledig mee eens

Casus 2

Patiënt X van **40 jaar, middelbaar opgeleid,** komt bij u op het spreek uur voor een **diabetes** controle. De patiënt ervaart **hier geen symptomen van**. U heeft **geen goede behandelrelatie** met de patiënt. De patiënt **heeft onvoldoende kennis van de ziekte** en heeft een **niet realistische ziekte-perceptie**. Er is **sociale steun thuis**. De patiënt **is niet gemotiveerd voor zelfmanagement.** De patiënt heeft **weinig vertrouwen in eigen kunnen**. Patiënt heeft **geen angst of stemmingsstoornis.**

Casus 3

Patiënt X van **40 jaar, hoog opgeleid,**  komt bij u op het spreekuur voor een **diabetes** controle. De patiënt ervaart **hier milde symptomen van**. U heeft een **goede behandelrelatie met de patiënt**. De patiënt **heeft voldoende kennis van de ziekte** en heeft een **realistische ziekte-perceptie**. Er is **sociale steun thuis**. De patiënt **is gemotiveerd voor zelfmanagement**. De patiënt heeft **voldoende vertrouwen in eigen kunnen**. De patiënt heeft **geen angst of stemmingsstoornis**.

Casus 4

Patiënt X van **80 jaar, laag opgeleid**, komt bij u op het spreek uur voor een **COPD** controle. De patiënt ervaart **hier milde symptomen van**. U heeft **een goede behandelrelatie** met de patiënt. De patiënt **heeft voldoende kennis van de ziekte** en heeft een **realistische ziekte-perceptie**. Er is **geen sociale steun thuis**. De patiënt **is gemotiveerd voor zelfmanagement**. De patiënt heeft **weinig vertrouwen in eigen kunnen**. Patiënt heeft **geen angst of stemmingsstoornis**.

Casus 5

Patiënt X van **80 jaar, laag opgeleid**, komt bij u op het spreek uur voor een **COPD** controle. De patiënt ervaart **hier geen symptomen van**. U heeft **geen goede behandelrelatie** met de patiënt. De patiënt **heeft onvoldoende kennis van de ziekte** en heeft een **niet realistische ziekte-perceptie**. Er is **sociale steun thuis**. De patiënt **is niet gemotiveerd voor zelfmanagement.** De patiënt heeft **voldoende vertrouwen in eigen kunnen**. Patiënt heeft **een angst of stemmingsstoornis**.

Casus 6

Patiënt X van **60 jaar, middelbaar opgeleid**, komt bij u op het spreekuur voor een **diabetes** controle. De patiënt ervaart **hier ernstige symptomen van**. U heeft een **goede behandelrelatie met de patiënt**. De patiënt **heeft voldoende kennis van de ziekte** en heeft een **realistische ziekte-perceptie**. Er is **geen sociale steun thuis**. De patiënt **is gemotiveerd voor zelfmanagement**. De patiënt heeft weinig **vertrouwen in eigen kunnen**. De patiënt heeft **geen angst of stemmingsstoornis**.

Casus 7

Patiënt X van **60 jaar**, **laag opgeleid**, komt bij u op het spreekuur voor een **COPD** controle. De patiënt ervaart **hier milde symptomen van.** U heeft een **goede behandelrelatie met de patiënt.** De patiënt **heeft onvoldoende kennis van de ziekte** en heeft een **niet realistische ziekte-perceptie**. Er **is** **sociale steun thuis**. De patiënt **is niet gemotiveerd voor zelfmanagement.** De patiënt heeft voldoende **vertrouwen in eigen kunnen**. De patiënt heeft **een angst of stemmingsstoornis**.

Casus 8

Patiënt X van **80 jaar, laag opgeleid**, komt bij u op het spreek uur voor een **diabetes** controle. De patiënt ervaart **hier milde symptomen van**. U heeft **een goede behandelrelatie** met de patiënt. De patiënt **heeft onvoldoende kennis van de ziekte** en heeft een **niet realistische ziekte-perceptie**. Er is **geen sociale steun thuis**. De patiënt **is niet gemotiveerd voor zelfmanagement**. De patiënt heeft **weinig vertrouwen in eigen kunnen**. Patiënt heeft **geen angst of stemmingsstoornis**.

Casus 9

Patiënt X van **60 jaar, hoog opgeleid**, komt bij u op het spreek uur voor een **diabetes** controle. De patiënt ervaart **hier ernstige symptomen van**. U heeft **geen goede behandelrelatie** met de patiënt. De patiënt **heeft voldoende kennis van de ziekte** en heeft een **realistische ziekte-perceptie**. Er **is sociale steun thuis**. De patiënt **is gemotiveerd voor zelfmanagement**. De patiënt heeft **weinig vertrouwen in eigen kunnen**. Patiënt heeft **een angst of stemmingsstoornis**.

Casus 10

Patiënt X van **60 jaar, middelbaar opgeleid**, komt bij u op het spreek uur voor een **COPD** controle. De patiënt ervaart **hier ernstige symptomen van**. U heeft **een goede behandelrelatie** met de patiënt. De patiënt **heeft voldoende kennis van de ziekte** en heeft een **realistische ziekte-perceptie**. Er **is sociale steun thuis**. De patiënt **is niet gemotiveerd voor zelfmanagement**. De patiënt heeft **weinig vertrouwen in eigen kunnen**. Patiënt heeft **geen angst of stemmingsstoornis**.

Casus 11

Patiënt X van **80 jaar, laag opgeleid**, komt bij u op het spreek uur voor een **diabetes** controle. De patiënt ervaart **hier geen symptomen van**. U heeft **geen goede behandelrelatie** met de patiënt. De patiënt **heeft onvoldoende kennis van de ziekte** en heeft een **niet realistische ziekte-perceptie**. Er is **geen sociale steun thuis**. De patiënt **is niet gemotiveerd voor zelfmanagement**. De patiënt heeft **voldoende vertrouwen in eigen kunnen**. Patiënt heeft **een angst of stemmingsstoornis**.

Casus 12

Patiënt X van **60 jaar, hoog opgeleid**, komt bij u op het spreek uur voor een **COPD** controle. De patiënt ervaart **hier geen symptomen van.** U heeft **geen goede behandelrelatie** met de patiënt. De patiënt **heeft onvoldoende kennis van de ziekte** en heeft een **niet realistische ziekte-perceptie**. Er is **geen sociale steun thuis**. De patiënt **is gemotiveerd voor zelfmanagement**. De patiënt heeft **voldoende vertrouwen in eigen kunnen**. Patiënt heeft **geen angst of stemmingsstoornis**.
