# Supplementary figures and images for: Patient factors that influence clinicians’ decision making in self-management support: A clinical vignette study
Source: PLoS One. 2017 Feb 6;12(2):e0171251. doi: 10.1371/journal.pone.0171251 (PMC5293247; doi:10.1371/journal.pone.0171251)

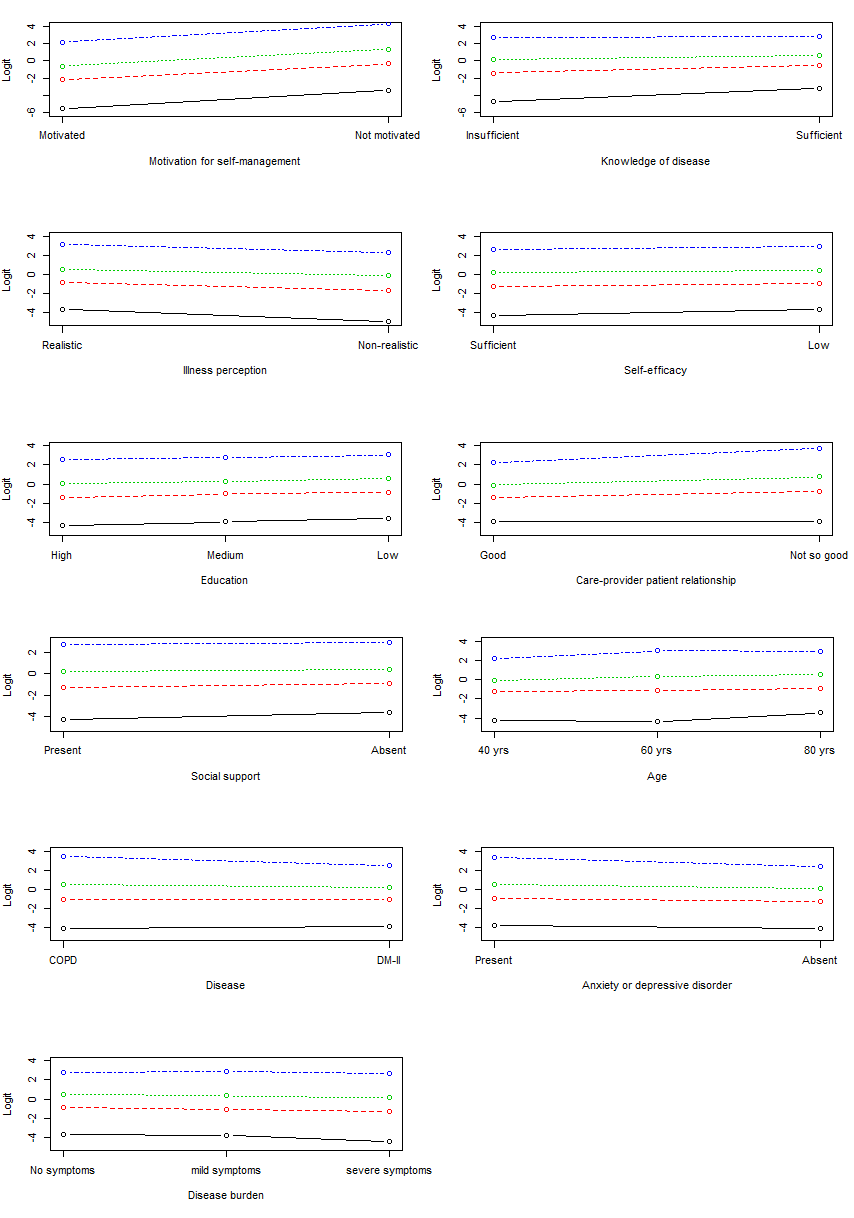

Supplement: S1 Fig — The lines in the graph represent the different dichotomized models (solid line represents the model unlikely to very likely to provide SMS vs. very unlikely to provide SMS, and the dashed line represents the model somewhat likely to very likely to provide SMS vs unlikely and very unlikely to provide SMS, etc., for the dotted and the dashed line). (TIF) [file pone.0171251.s002.tif]
