# Supplementary material for: Patient factors that influence clinicians’ decision making in self-management support: A clinical vignette study
Source: PLoS One. 2017 Feb 6;12(2):e0171251. doi: 10.1371/journal.pone.0171251 (PMC5293247; doi:10.1371/journal.pone.0171251)
Supplement: S1 Table — SM = Self-management, CI = confidence interval, GP = general practitioner, PN = practice nurse, DM-II = Diabetes mellitus type II, COPD = Chronic obstructive pulmonary disease. (DOCX) [file pone.0171251.s003.docx]

|  |  | Providing SM support  Log likelihood(CI) | Patient will be successful  Log likelihood(CI) | | |
| --- | --- | --- | --- | --- | --- |
| Motivation | Total | 218.1 | 288.8 |  |  |
|  | GP | 90.9 (68.9-113.9) | 137.5 (110.4-171.0) | |  |
|  | PN | 127.6 (102.0-160.0) | 151.1 (125.6-183.9) | | |
| Patient-provider relationship | Total | 54.3 | 68.5 | | |
|  | GP | 15.1 (6.5-28.7) | 26.7 (14.4-42.4) | | |
|  | PN | 41.1 (25.6-62.4) | 43.7 (26.5-64.3) | | |
| Illness perception | Total | 25.3 | 35.5 | | |
|  | GP | 22.3 (11.0-38.1) | 28.8 (16.8-44.5) | | |
|  | PN | 6.8 (1.5-16.5) | 10.5 (3.5-22.6) | | |
| Having a depression or anxiety disorder | Total | 19.8 | 23.0 | | |
|  | GP | 10.6 (3.4-22.5) | 8.2 (2.3-18.0) | | |
|  | PN | 10.0 (3.3-20.8) | 15.1 (7.1-27.9) | | |
| Education level | Total | 16.1 | 14.8 | | |
|  | GP | 8.6 (2.6-20.7) | 7.6 (2.5-18.3) | | |
|  | PN | 8.8 (2.8-20.3) | 7.5 (2.2-18.2) | | |
| Self-efficacy | Total | 13.6 | 21.4 | | |
|  | GP | 7.1 (1.3-17.6) | 9.0 (2.5-20.5) | | |
|  | PN | 7.4 (1.5-16.7) | 12.4 (4.3-24.7) | | |
| Social support | Total | 11.4 | 17.9 | | |
|  | GP | 4.3 (0.4-13.1) | 7.2 (1.7-17.3) | | |
|  | PN | 6.9 (1.3-15.9) | 10.8 (3.5-22.2) | | |
| Age | Total | 7.3 | 2.8 | | |
|  | GP | 5.4 (1.1-15.4) | 2.1 (0.2-8.6) | | |
|  | PN | 2.8 (0.2-10.7) | 1.3 (0.1-7.1) | | |
| Disease severity | Total | 6.4 | 7.5 | | |
|  | GP | 8.4 (2.6-20.2) | 7.2 (1.9-18.6) | | |
|  | PN | 1.3 (0.1-7.4) | 1.9 (0.0-8.5) | | |
| Knowledge of disease | Total | 3.2 | 6.8 | | |
|  | GP | 1.6 (0.01-7.1) | 2.9 (0.1-10.3) | | |
|  | PN | 1.8 (0.0-8.4) | 3.8 (0.1-11.9) | | |
| Disease DM-II vs COPD | Total | 0.5 | 2.6 | | |
|  | GP | 0.1 (0.0-3.4) | 0.8 (0.0-6.3) | | |
|  | PN | 0.5 (0.0-5.1) | 1.7 (0.1-8.8) | | |
